# Multivariable analysis to determine risk factors associated with abortion in mares

**DOI:** 10.1530/RAF-22-0087

**Published:** 2022-11-14

**Authors:** J M Roach, J C Arango-Sabogal, K C Smith, A K Foote, K L Verheyen, A M de Mestre

**Affiliations:** 1 Comparative Biomedical Sciences, Royal Veterinary College, Hawkshead Lane, Hatfield, UK; 2 Pathobiology and Population Sciences, Royal Veterinary College, Hawkshead Lane, Hatfield, UK; 3 Rossdales Laboratories, Newmarket, UK

**Keywords:** mare, abortion, pregnancy, horse, risk factor

## Abstract

Risk factors associated with equine reproductive efficiency have been identified along with those associated specifically with early pregnancy loss (EPL). In contrast, no studies have reported risk factors associated with abortion (loss between days 70 and 300 post-cover). Given the causes of abortion differ from those of EPL, likely too will the risk factors. A retrospective cohort study was carried out to identify risk factors associated with abortion in UK- and Irish-based Thoroughbreds, collecting data on 20 exposure variables over a 5-year period. A generalized linear mixed model was utilized to evaluate the associations between exposure variables and abortion, with clustering of observations accounted for at the mare and farm level. Variables with a likelihood ratio test (LRT) *P* value < 0.2 were entered into the model in a forward stepwise approach. Pregnancy outcome was available on 4439 pregnancies from 2510 mares. Having had two or more prior abortions (odds ratio (OR): 7.91, 95% CI: 2.86, 21.88), conceiving on the second or subsequent covered estrous cycle (OR: 1.84, 95% CI: 1.22, 2.78) and conceiving multiple conceptuses (OR: 1.68, 95% CI: 1.02, 2.76) were associated with an increased risk of abortion compared to null parous, first estrous cycle covers and singleton conceptions, respectively. Increasing paternal age (OR: 0.95, 95% CI: 0.90, 0.99) was associated with a decreasing risk of abortion. Mare and farm variance were not significant in the final model, LRT *P* = 0.43. These findings provide evidence-based data to inform Thoroughbred breeding management practices to help mitigate abortion risk.

**Lay summary:**

This is the first study to identify the risk factors (characteristics which change the chance of an event) for abortion (miscarriage between days 70 and 300 of pregnancy) in the horse. Statistical models were used to account for the interactions between 20 different factors. The factor which increased the mare’s risk of having an abortion the most was when she had had two or more abortions prior to the pregnancy. Additionally, when the mare was initially pregnant with twins but one of those pregnancies was reduced, the remaining pregnancy was at an increased risk of aborting. Older mares were not at an increased risk of abortion like in humans; however, pregnancies fathered by older stallions were less likely to abort than those from younger stallions. The findings of this study can inform horse breeding practices to help reduce the chance of an abortion.

## Introduction

Equine pregnancies can fail at any stage of gestation attributed to both intrinsic and extrinsic factors. The incidence risk of pregnancy failure after day 70 in gestation has not improved in almost 20 years (Allen *et al.* 2007, Rose *et al.* 2018), with 7.3% of day 70 pregnancies failing to produce a live foal (Roach *et al.* 2021). Most of these pregnancy failures were abortions (54.9%), defined as a loss occurring between days 70 and 300 of gestation. Whilst several studies have identified risk factors for pregnancy loss prior to day 65 of gestation (Allen *et al.* 2007, Hanlon *et al.* 2012, Rose *et al.* 2018, Roach *et al.* 2021), very little research has been carried out to determine risk factors for pregnancy losses occurring later in gestation.

Several mare and stallion risk factors have been associated with reproductive performance either through efficiency of conception (Allen & Wilsher 2012, Hanlon *et al.* 2012, Lane *et al.* 2016) or early pregnancy loss (EPL) (Nath *et al.* 2010, de Mestre *et al.* 2019). Risk factors for pregnancy loss throughout later gestation have also been explored (Chevalier-Clément 1989, Allen *et al.* 2007, Bosh *et al.* 2009, Miyakoshi *et al.* 2012); however, these studies have often used wide periods of time (e.g. ‘day 35 to foaling’ (Miyakoshi *et al.* 2012)) and aside from Miyakoshi *et al*., only employed univariable analysis. Very few studies have explored risk factors specific to pregnancy losses after the first 2 months of gestation and none, to the author’s knowledge, have investigated equine abortion exclusive of late EPLs (42–65 days of gestation) and stillbirths as an overt phenotype. Given the differences in the underlying causes of failure to conceive, EPL, abortion and stillbirth, investigation of phenotype-specific risk factors is warranted.

Maternal factors affecting wider reproductive success have been identified from conception onwards, with increasing mare age and barren or slipped mares exhibiting lower pregnancy rates compared to their younger, maiden counterparts (Allen *et al.* 2007, Bosh *et al.* 2009, Nath *et al.* 2010, Hanlon *et al.* 2012, Lane *et al.* 2016). Multivariable analysis suggests, in addition to mare age, that it was the mare having had one previous live foal, as opposed to status, which was associated with an increased risk of EPL (Roach *et al.* 2021). Whilst late pregnancy loss increased in mares over the age of 20 years old in one study (Chevalier-Clément 1989), neither mare age nor status was associated with an increased risk of pregnancy loss after day 35 of gestation using multivariable analysis (Miyakoshi *et al.* 2012). Contradictory findings have been reported as to covering a mare on foal heat, with both a significant increase in risk of not conceiving when covered on foal heat (Lane *et al.* 2016), and a significant positive association with foal heat cover and season pregnancy rates reported (Hanlon *et al.* 2012). Multivariable analysis found either no (de Mestre *et al.* 2019) or an increased risk of EPL with foal heat cover (Miyakoshi *et al.* 2012).

Multiple conceptions have been associated with a decreased risk of EPL (Nath *et al.* 2010). Whilst this was not the case in de Mestre *et al*.’s study (de Mestre *et al.* 2019), administration of flunixin meglumine at the time of twin reduction did reduce the risk of an EPL. A twin pregnancy maintained to later gestation is strongly associated with abortion (Weber *et al.* 2018), but whether a reduced multiple conception pregnancy is at an increased risk beyond 70 days is not known. The administration of progesterone has not been associated with a change in risk of pregnancy loss (Miyakoshi *et al.* 2012); however, the length and indications for treatment were not detailed in the study. The extent to which therapeutic interventions modify the risk of abortion is currently poorly understood.

The influence of stallion factors cannot be overlooked when reporting on risk factors associated with reproductive efficiency. Both Bosh *et al.* in the US (Bosh *et al.* 2009) and Allen *et al*. in the UK (Allen & Wilsher 2012) explored the effect of a stallion’s book size (the number of mares a stallion is assigned to cover in that breeding season) on conception, with neither reporting a significant decline in fertility with increasing book size. Data from Ireland, however, found that very high stallion usage, defined as greater than 21 covers in a week, was associated with lower pregnancy rates (Lane *et al.* 2016). Allen *et al*.’s study (Allen & Wilsher 2012) did find that the per-mating pregnancy rate was significantly lower following 1 or more days of sexual rest compared to those where a mating had occurred at least once the day preceding, suggesting that it is the frequency of breeding rather than book size which may be significant (Allen *et al.* 2007). Shuttled stallions (stallions that bred mares in the Southern hemisphere the season prior) have also been shown to have lower per-cycle pregnancy loss rates, perhaps reflecting a healthy worker effect or improved management of those individuals (Lane *et al.* 2016).

In human medicine, risk factors for miscarriage (failure of the pregnancy prior to extrauterine viability) have been explored in detail. Increased maternal age is a major, independent risk factor for loss (Coste *et al.* 1991, Quenby *et al.* 2021), as is seen for EPL in the mare, although this association disappeared in one study when losses due to congenital abnormalities were excluded (Gardosi *et al.* 2013). Similarly, a paternal age of over 40 years is a risk factor for miscarriage, and this is independent of the maternal age (Quenby *et al.* 2021).Coste *et al.* (1991) found that gravidity and parity were not associated with loss; however, they would likely not have seen the grand multiparity which can occur in Thoroughbreds. Previous miscarriage is a major risk factor for subsequent losses, with the risk increasing by around 10% for each subsequent miscarriage (Quenby *et al.* 2021). Whilst anecdotally mares with a history of pregnancy loss are thought to be at greater risk, there is little data supporting or quantifying this assumption.

Risk factors associated with reproductive efficiency, conception and foaling have been reported, along with multiple studies identifying those associated specifically with EPLs. Despite the significant differences in underlying causes of infertility in the mare, fewer studies have investigated risk factors for specific phenotypes associated with failure to produce a live offspring and none for abortion. We recently quantified the causes of abortion in the mare (Roach *et al.* 2021) which highlighted stark differences in causes when compared with failure to conceive and EPL. Therefore, the aim of this study was to identify risk factors associated with abortion (pregnancy loss between gestation days 70 and 300) in a large cohort of UK- and Irish-based Thoroughbreds.

## Materials and methods

### Study design and population

A retrospective cohort study collected data from five breeding seasons (2013–2017). A convenience sample of stud farms, located in the Southeast of England and Ireland, was recruited. Mares were included in the study if they were Thoroughbred, covered by a UK- or Ireland-based stallion and pregnant at day 70 post cover. Day 70 of gestation was chosen, to exclude losses diagnosed at the day 65 scans which were likely a result of pathologies established during the early pregnancy period (Rose *et al.* 2018).

### Ethical considerations

The Royal Veterinary College’s Clinical Research Ethical Review Board granted ethical approval: URN 2017 1660-3. Written informed consent was obtained from all participating farms and data were coded for anonymity.

### Data collection

The data collection of exposures over the 2013 and 2014 seasons was described in detail by Rose *et al.* (2018) and de Mestre *et al.* (2019). Briefly, a custom Access (Microsoft Access for Office 365, 2019 build, Microsoft) database was built to input the data, with questionnaires utilized for on-farm data collection (Rose *et al.* 2018). The 2013 and 2014 exposure data were exported into an Excel workbook (version 2016, Microsoft) and the pregnancy outcome supplemented, along with the cause and date of pregnancy loss when necessary. The data from seasons 2015 to 2017 were inputted directly into the Excel workbook.

### Exposure variables

Stud records were supplemented with veterinary practice records, Weatherbys published data (the UK Thoroughbred General Studbook, Weatherbys Return of Mares (ROM) (Weatherbys 2013*b*, 2014b, 2015*b*, 2016*b*, 2017*b*, 2018*b*), The Weatherbys Stallion Books (Weatherbys 2013*a*, 2014*a*, 2015*a*, 2016*a*, 2017*a*, 2018*a*) and online publicly available databases (Racing Post database (www.racingpost.co.uk) and Arion Pedigrees (https://www.arion.co.nz)). Supplementary data were provided by Weatherbys including import/export dates and mare covering and produce records. Data were collected on a total of 20 variables: mare- and stallion-identifying (ID) codes along with their respective farms and then eight mares, three stallions, three pregnancies and two extrinsic factors.

Mare factors collected were age of the mare, status at the start of the breeding season, date of cover, age to stud, number of previous live foals, number of previous abortions, total number of years covered and the number of estrous cycles that the mare was covered on. Mare status was categorized as maiden (never bred); foaling (pregnant at the start of the season); barren (not pregnant at the start of the breeding season) or rested (not bred the season prior but covered previously). Data provided by Weatherbys allowed collection of exposure variables related to the mare’s prior reproductive history. This included the number of previous abortions a mare had suffered, defined as the number of years the mare had a registered abortion event excluding the current pregnancy, and the number of previous foals the mare had produced, defined as the number of years the mare produced a live foal to registration, up to and including the foal born the year of cover. The number of active seasons where the mare was booked to a stallion was also recorded. Lastly, the year on which the mare was registered as a broodmare in the UK was recorded to calculate the variable ‘age to stud’.

Data on stallion factors, in addition to stallion- and stallion farm-ID, included the age of the stallion at the time of cover, the stallion’s book size and whether the stallion had covered mares in the Southern hemisphere the season prior (‘shuttle status’). Arion Pedigrees and the Weatherbys Stallion books provided data on the stallion age, residing stud and shuttle status. Weatherbys ROM data were used to record the book size of the covering stallion, defined as the total number of mares the stallion was booked to go to that season (Northern hemisphere only).

Pregnancy factors were recorded using the stud and veterinary clinical records. These exposures included fetal sex, whether the pregnancy derived from a reduced multiple conception and whether the mare was given altrenogest (Regumate® Equine 2.2 mg/mL oral solution MSD Animal Health) at any stage of gestation following a positive pregnancy diagnosis.

The year of cover was recorded as an extrinsic factor and the locations of the mare and stallion farms were compared to identify mares that would have traveled following covering (mare from a UK farm bred to a stallion standing in Ireland and vice versa).

### Outcome

Stud records were used to record the pregnancy outcome. When the pregnancy outcome was not available through the stud records, the Weatherbys ROM data were used. Pregnancies were excluded when no foaling outcome was available, no date of loss was recorded or in cases of mare death during gestation.

### Statistical analysis

Statistical analyses were conducted in Stata statistical software (Release 16;Statacorp).

### Descriptive analyses

The proportion of pregnancies resulting in abortion was calculated for each level of the exposure variables, along with the 95% CI. The normality of continuous variables was assessed both visually and using the Shapiro–Wilk test for normality, with the mean and s.d. or median, interquartile range (IQR) and range reported as appropriate. Proportions and 95% CI were reported for categorical data.

### Risk factor analyses

The linearity of continuous variables and the log-odds of the outcome were assessed using Stata’s ‘lintrend’ command (Garrett 2017). If linearity was not respected, variables were categorized into quartiles or in line with previous literature (de Mestre *et al.* 2019).

The number of estrous cycles the mare was covered on was grouped into pregnancies conceived on the first or a subsequent estrous cycle. The number of previous abortion events the mare had was categorized into maiden, mare previously bred but never aborted, one previous abortion and two or more previous abortions. The age the mare entered stud was grouped into those entering stud prior to 4 years of age, those entering between 4 and 7 years old and then those entering at 8 years old or greater.

Book size was categorized based on the average number of covers that it would approximately equate to per day. Assuming an average conception rate of 60% (Morris & Allen 2002, Allen & Wilsher 2012) and a breeding season of 120 days, the stallions were categorized as breeding up to, on average, every other day (book size ≤ 35 mares), once a day (36–72 mares), twice a day (73–145 mares), 3 times a day (146–218) and more than 3 times a day (≥219 mares).

### Univariable analysis

Univariable analysis was carried out to assess the relationship between the exposure variables and the outcome, abortion. A generalized linear mixed model (GLMM) was used, accounting for clustering at the mare and mare farm level. Exposure variables with a univariable likelihood ratio test (LRT) *P* value < 0.2 were taken forward for multivariable analysis.

### Multivariable analysis

Variables were entered into the model in a forward stepwise approach and retained when the LRT *P* value was <0.05. Two-way interactions were explored between all exposure variables retained in the final model using Wald tests and retained if the *P* value was <0.05. Confounding between the number of prior abortions with mare age and, separately, with the total number of years covered was explored by forcing the variables into the model and assessing the change in coefficients. A change greater than 20% in the coefficient signaled that the forced variable should be retained.

To check that the assumptions of GLMM model were met, the normality of the residuals and their homoscedasticity at the highest level (farm) were visually assessed by using Q-Q plots and plots of residuals against the predicted values.

## Results

### Descriptive analysis results

#### Population descriptive results

Data were collected on 4639 pregnancies. The pregnancy outcome was available for 95.7% of the pregnancies (*n* = 4439) from 2510 mares. The remaining 200 pregnancies were excluded due to either no pregnancy outcome being available (*n* = 113), the mare dying during gestation (*n* = 27) or the pregnancy failing but no day of loss being recorded (*n* = 50).

Data were collected from 29 farms although not all farms contributed data for all years. Data were available from 27 farms in 2013, 23 in 2014 and 8 for seasons 2015–2017. Data were collected on 1145 pregnancies in 2013, 957 in 2014, 762 in 2015, 787 in 2016 and 788 in 2017.

The median number of pregnancies per mare was 1 (IQR: 1–2, range: 1–5), with 44.1% of the mares (*n* = 1107) having repeated observations. The median mare age at pregnancy was 8 years old (IQR: 5–11, range: 3–24). Of the pregnancies, 17.1% (*n* = 761) were from maiden mares, 65.4% (*n* = 2904) were from foaling mares, 13.3% (*n* = 592) were from barren mares and 4.1% (*n* = 182) were from rested mares. There were 142 stallions included in the study, residing on 33 farms. The median number of pregnancies per stallion was 9 (IQR: 3–37, range: 1–459), with 83.1% (*n* = 118) having repeated observations. The median stallion age was 11 years old (IQR: 7–14, range: 3–25).

### Exposure variables descriptive results

A description of the exposure variables investigated along with the proportion of cases which aborted for each level is presented in Supplementary data 1 (see section on supplementary materials given at the end of this article). Only one variable, administration of altrenogest during gestation, had greater than 25% missing data. The proportion of missing data for each exposure variable is summarized in Supplementary data 2.

## Risk factors associated with abortion

### Outcome

There were 163 cases of abortion and 4276 pregnancies surviving past day 300 of gestation, resulting in an overall incidence of abortion of 3.7% (95% CI: 3.1–4.3) between days 70 and 300 of gestation.

### Univariable analysis results

The univariable analysis results for risk factors associated with abortion are presented in Supplementary data 3.

### Multivariable analysis results

Six mare factors, one stallion factor, one pregnancy and one extrinsic factor were taken forward for multivariable modelling. The mare factors were mare age, mare status, the number of previous abortions the mare had suffered, whether the mare conceived the pregnancy on her first or subsequent breeding cycle, the number of live foals the mare had produced to date and the total number of years the mare had been covered. The pregnancy and extrinsic factors were whether the pregnancy was derived from a twin conception and the year of cover, respectively. Lastly, the age of the covering stallion was taken forward.

The final model for risk factors associated with abortion in this cohort of pregnancies is presented in Table 1. The variance of the mare and mare farm were not significant, *P* = 0.43 (mare variance 0.12 (CI: 0.0–5207.08) and farm variance zero (no CI)).

**Table 1:** Final generalised linear mixed model of risk factors associated with abortion (pregnancy loss between day 70 and 300 of gestation) in a cohort of 1,709 Thoroughbred mares from 25 farms over the 2013-2017 UK breeding seasons (2,571 pregnancies).

Predictor / Category	Abortion cases, *n*	Total *n*	Odds ratio	95% CI	Wald *P* value	LRT* *P* value
Previous abortions						0.002
Maiden	17	596	Reference			
Bred, never aborted	69	1,766	1.26	0.73, 2.18	0.41	
One previous abortion	10	177	1.65	0.72, 3.78	0.23	
Two or more abortions	7	32	7.91	2.86, 21.88	<0.001	
Stallion age (years)	103	2571	0.95	0.90, 0.99	0.014	0.012
Pregnant on first estrous cycle						
Yes	59	1833	Reference			
No	44	738	1.84	1.22, 2.78	0.004	0.005
Conceptus						
Singleton	80	2185	Reference			
Multiple conceptus	23	386	1.68	1.02, 2.76	0.04	0.047

*Likelihood ratio test, mare variance and farm variance *P* = 0.43.

There was no evidence of confounding between the number of previous abortions the mare had had and the age of the mare and, separately, the total number of years the mare had been covered, to account for ‘time at risk’; therefore, neither variable was forced into the model. To understand whether there was an ‘aged genetics’ effect on abortion, confounding between mare age and stallion age was evaluated but no evidence was found (coefficients changed by <0.001% with the addition of mare age); therefore, again, mare age was not retained in the final model.

The strongest association with abortion was in mares who had suffered two or more abortions compared to maiden mares (OR: 7.91; 95% CI: 2.86, 21.88). This significant increase in odds was consistent when comparing mares that had suffered two or more previous abortions to mares that had been bred but never aborted (OR: 6.29; 95% CI: 2.50, 15.79) and to mares that had suffered one previous abortion (OR: 4.78; 95% CI: 1.56, 14.80), *P* <  0.001 and *P* = 0.006, respectively. There was a significant 5% decrease in the odds of the pregnancy resulting in an abortion for each year increase in the age of the stallion siring the pregnancy (O: 0.95; 95% CI: 0.90, 0.99). Pregnancies which were not conceived on the first estrous cycle the mare was covered on were 84% more likely to end in abortion (OR: 1.84; 95% CI: 1.22, 2.78) than those conceived on the first estrous cycle covered on. Lastly, pregnancies which were originally a multiple conception and subsequently reduced were 68% more likely to be aborted compared to singleton conceptions (OR: 1.68; 95% CI: 1.02, 2.76). No significant interactions were observed in the final model.

**Figure 1:**
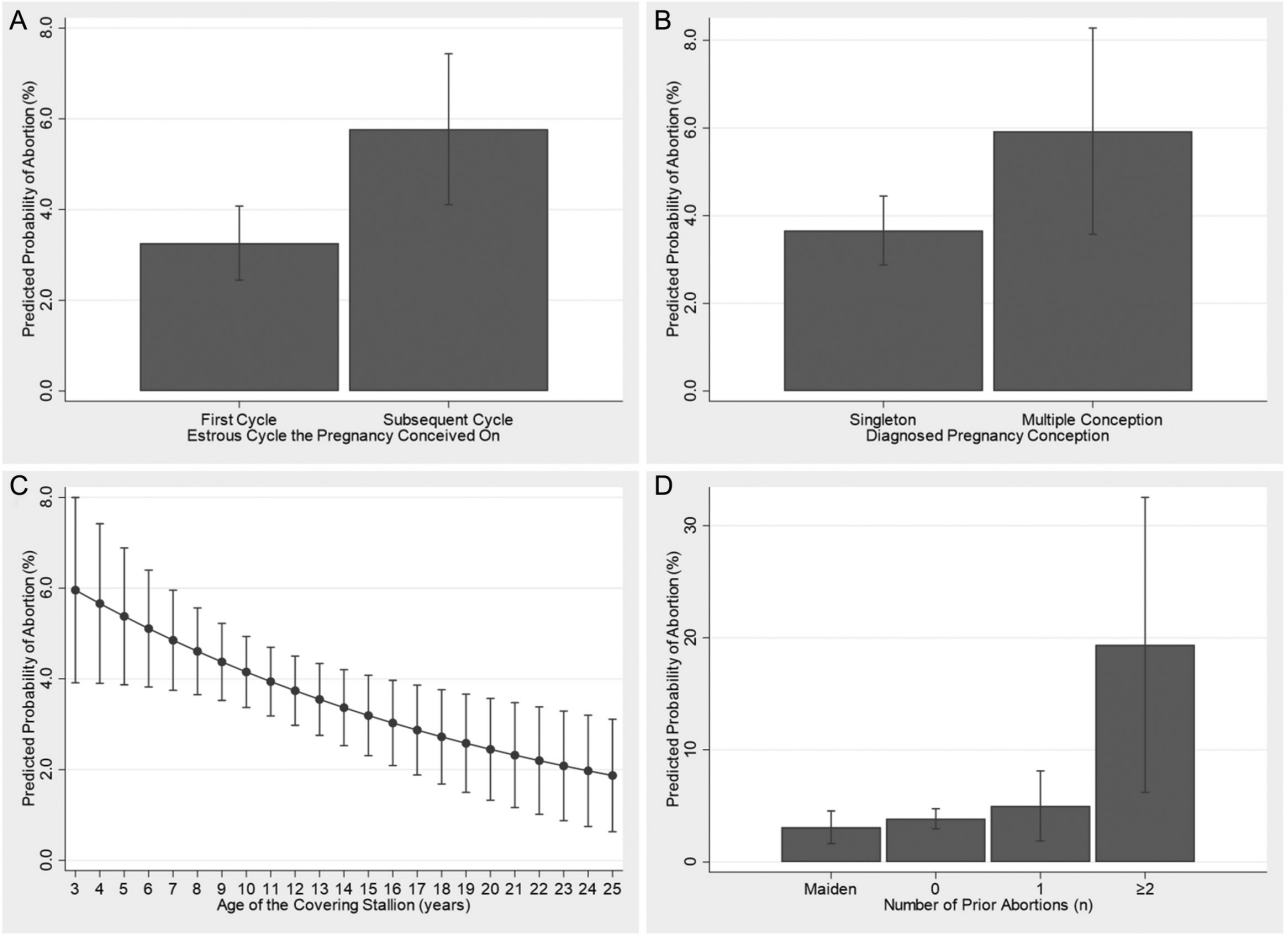
Predicted probability of abortion and 95% CIs between (A) estrous cycle the pregnancy was conceived on, (B) diagnosed pregnancy conception, (C) age of the covering stallion and (D) number of prior abortions. Estimates were obtained from the generalized linear mixed model evaluating risk factors associated with abortion in UK Thoroughbred pregnancies (*n* = 2571).

For illustrative purposes, the predicted probability of the outcome estimated from the final model for each retained exposure is presented in Fig. 1A, B, C and D. The predicted probability of abortion for the estrous cycle the mare was covered on (Fig. 1A) and whether the pregnancy was derived from a multiple conceptus or not (Fig. 1B) illustrate that the increase in the probability of abortion for each factor almost doubles from 3.3 to 5.8% and 3.7 to 5.9%, respectively. The predicted probability of abortion across stallion age (Fig. 1C) ranges from 6.0% for a 3-year-old covering stallion to 1.9% for a 25-year-old stallion, with a linear decline. The population data and model were used to illustrate the predicted probability of abortion dependent on the number of previous abortions a mare has had (Fig. 1D). Finally, Fig. 1D illustrates that the probability of a pregnancy resulting in abortion was 19.4% when carried in a mare who had suffered two or more abortions, compared to 3.7% in the general population.

## Discussion

This retrospective study of over 2500 pregnancies details risk factors associated with abortion in the horse. Four factors led to a modified risk of abortion in this population, three of which were unique to abortion having not been described for either failure to conceive or EPL. The strongest increase in the risk of abortion was seen in mares who had suffered two or more previous abortions. Pregnancies that were not conceived on the first estrous cycle the mare was covered on as well as pregnancies derived from now reduced multiple conceptions were approximately twice as likely to abort compared to those conceived on the first cycle and singleton conceptions. In contrast to the known effects of age on stallion breeding efficiency (Dowsett & Pattie 1982, Blanchard *et al.* 2010, Turner 2019) and human paternal aging effects (Nybo Andersen *et al.* 2004, Slama *et al.* 2005, du Fossé *et al.* 2020), increasing age of the covering stallion was associated with a decreasing risk of abortion. Unlike prior breeding efficiency and EPL studies (Bosh *et al.* 2009, Lane *et al.* 2016, de Mestre *et al.* 2019), neither the age of the mare nor parity was associated with a modified risk of abortion. The findings of this study provide evidence-based data to inform breeding and clinical management practices to mitigate abortion risk.

A mare having had two or more prior abortions represented the most significant exposure, leading to an almost eight-fold increase in the chance of the pregnancy aborting compared to a pregnancy in a maiden mare. This observation was limited to mares suffering two or more abortions, with no increased risk identified when only one prior abortion had occurred. The pathophysiology behind the significant increase in the risk of abortion following multiple, but not a single prior abortion is unclear. Possible explanations include an intrinsic factor such as a latent infection, for example, equine herpesvirus-1 (EHV-1) recrudescing in gestation (Lunn *et al.* 2009) and causing the abortion. However, we know that most pregnancy losses after day 70 of gestation are non-infectious in origin, with only 9.4% of pregnancy losses between day 70 of gestation and 24 h post parturition diagnosed as EHV (Roach *et al.* 2021). Alternative explanations for this increased risk include a predisposition through genetic variants that impact uterine and oocyte health, immunity or endocrine function or the presence of uterine disease. Given that umbilical cord torsion is the most significant cause of abortion in the UK Thoroughbred population (Roach *et al.* 2021), risk factors specific to umbilical cord torsion should be explored, with a genetic variant perhaps going some way to explaining this finding. The diagnosed cause of the prior abortions was not collated for this study but would have proved valuable in separating cases of repeat diagnoses from those where the diagnoses were unrelated.

Genetic causes of pregnancy loss have been described in early gestation; rare cases of chromosomal translocations in mares suffering recurrent pregnancy loss (Lear *et al.* 2008, 2014, Ghosh *et al.* 2016), or more commonly, aneuploidy (Shilton *et al.* 2020). Shilton *et al*. found 21.8% of EPLs to be aneuploid with preliminary data suggesting this is a genetic variant introduced during meiosis (Rizzo *et al.* 2020) rather than a random event of early blastocyst development (Shilton *et al.* 2020). There are limited descriptions of genetic variants associated with abortion in the mare (de Leon *et al.* 2012, Aurich *et al.* 2019, Dias *et al.* 2019). Inherited SNPs in the procollagen-lysine, 2-oxyglutarate 5-dioxygenase 1 gene can result in abortion and stillbirth, originally described in warmblood mares (Monthoux *et al.* 2015) but more recently also in a Thoroughbred (Grillos *et al.* 2022). A missense SNP in p53 gene in Thoroughbred mares in Brazil has also been found to be associated with an increased risk of abortion (de Leon *et al.* 2012). Based on the substantial increase in risk in repeat abortion mares, further work is warranted to identify other inherited genetic variants associated with abortion.

Sustaining a twin pregnancy through gestation is a known cause of abortion in the mare (Jeffcott & Whitwell 1973, Foote *et al.* 2012); however, the incidence of twin abortions is now negligible in intensively managed mares (Smith *et al.* 2003, Roach *et al.* 2021) due to the practice of manual reduction early in gestation. Chevalier-Clément’s 1989 study (Chevalier-Clément 1989) observed significantly higher abortion rates in twin conceptions, not only in those which received no treatment but also in those that underwent crushing of one of the twins. Additionally, although the rate of abortion in twin conceptions which underwent natural selective reabsorption following pregnancy diagnosis was still significantly higher than single conceptions, it was numerically lower than those that had an interventional crushing of the conceptus (Chevalier-Clément 1989). Whilst twin management has improved since Chevalier-Clément’s study, with no increased risk of EPL associated with a multiple conception (Nath *et al.* 2010, de Mestre *et al.* 2019), the observation in our multivariable analysis that a reduced twin pregnancy is still at an increased risk of abortion is an important finding. The potential pathways for an increased risk of abortion but not EPL are thought-provoking. Ginther reported that two-thirds of twin embryo pregnancies underwent spontaneous reduction between days 17 and 40 of gestation (Ginther 1984), and to date, we do not understand the mechanisms for intrinsic selection. With arbitrary decisions over which pregnancy to reduce such as vesicle size and conceptus location (Schnobrich *et al.* 2013), along with early intervention at days 14–16 of gestation, perhaps we are intervening prematurely and, in some cases, wrongly retaining the pregnancy which may have gone on to spontaneously reduce (Ginther 1984). Direct damage incurred to the surviving conceptus, or inadvertent disruption of the implantation location, through physical manipulation during twin reduction could represent an alternative cause of the increased risk of abortion. The location of implantation is vitally important for the ongoing health of the pregnancy, with those implanting in the caudal uterus shown to have a higher risk of pregnancy failure prior to day 42 of gestation (Jobert *et al.* 2005), and ‘body pregnancies’ a recognized cause of abortion (Smith *et al.* 2003, Foote *et al.* 2012). Additionally, following a twin reduction, there is a release of PGF2α in response to the localized uterine inflammation and operator-applied pressure (Pascoe *et al.* 1987), which has a luteolytic action. Flunixin meglumine, a prostaglandin synthetase inhibitor, is often administered (Rose *et al.* 2018) to counteract the luteolytic action and has been shown to decrease the risk of EPL following twin reductions (de Mestre *et al.* 2019), but its impact on abortion has not been explored here or elsewhere. Ultimately, the procedure of twin reduction may be disrupting the natural process of the conceptus moving around the uterus and contacting with the endometrium, modulating cytokine release, unintentional selection bias, and be associated with the use of additional therapeutics. Together, or individually, these may all be reflected in the observed increase in the risk of abortion in previously reduced multiple conception identified in this work. Therefore, the management of embryo pregnancy reductions warrants a review.

An increased risk of abortion was identified in the pregnancies that were not conceived on the first estrous cycle the mare was covered on. In this study, a mare not being pregnant on the first estrous cycle covered on could be a result of either failure of conception, early embryonic loss (EEL) prior to pregnancy diagnosis, or EPL between days 15 and 70. Work by Ball *et al.* estimated EEL to range from 0% to 9% of pregnancies in fertile young mares, but up to 73% of pregnancies in aged, subfertile mares (Ball *et al.* 1986, 1989). Failure to conceive on first estrous cycle in this current study may therefore be accounting for what has previously been thought of as a maternal age effect. EPL is reported to occur in 6.4% of pregnancies between days 15 and 42 of gestation (Rose *et al.* 2018). While EPL can occur after day 42 of gestation (Rose *et al.* 2018), this would likely result in a failure to return to estrous resulting in these pregnancies not being included in this cohort. Without accounting for earlier losses (EEL and EPL prior to day 42) in this population, we have produced an unintentional bias. Pregnancies which were conceived on a subsequent cycle will have, in part, reflected a level of subfertility in the mare, either through endometritis, aberrant immunity or endometrial degeneration, and risk factors specific for EPL will be tied up in that variable. Further data collection to separate out subsequent estrous covers due to EPL from those that failed to conceive would allow better characterization of this finding.

Increasing stallion age was associated with a decreasing risk of abortion. This contrasts with other pregnancy loss phenotype analyses which found no effect (Blanchard *et al.* 2010, Lane *et al.* 2016, de Mestre *et al.* 2019). Although a stallion age effect is not observed as a risk factor for EPLs or seasonal reproductive measures (Lane *et al.* 2016, de Mestre *et al.* 2019), decreased reproductive efficiency of the older breeding stallion has been evidenced (Dowsett & Pattie 1982, Blanchard *et al.* 2010, Turner 2019). In human studies, advanced paternal age is a known risk factor for pregnancy loss throughout gestation (Nybo Andersen *et al.* 2004, Slama *et al.* 2005, du Fossé *et al.* 2020), with the risk at its highest for losses after 20 weeks of gestation (Nybo Andersen *et al.* 2004). One hypothesis for the decrease in risk of abortion associated with increasing stallion age would be selection bias in the population leading to a ‘healthy worker effect’ (Ogle 1885). The initial selection of a stallion for a career in breeding is not based solely on their fertility, but a complex combination of their race performance, pedigree and popularity (Colenbrander *et al.* 2003). Whilst reproductive soundness exams are performed (Hurtgen 1992), stallions do not need proven fertility to attract large numbers of mares for their initial seasons. The stallions that perform well in the shed and whose offspring receive good sales prices and perform well on the track will have a longer career well into their late teens and early 20s. A higher-than-average risk of abortion was predicted in covering stallions aged 3–9 years old compared to that of the observed population, which will include first- and second-season stallions with unproven fertility. Additionally, there may be a bias in the caliber of mares going to different aged stallions based on their covering fees which should be explored further.

No association was found between stallion book size and abortion in the current study. There are no previous reports on the effect of stallion book size on abortion as a phenotype. However, previous research has explored the effects on other measures of fertility. Allen and Wilsher (2012) looked at per-mating and seasonal pregnancy rates and found no decline in fertility in the stallions with large book sizes whilst Blanchard *et al.* (2010) found a positive correlation with seasonal pregnancy rates. In contrast,Walbornn *et al.* (2017) demonstrated a negative effect of increasing book size on a stallion’s first cycle- and per-cycle pregnancy rates. This effect was most prominent in novice stallions with the authors hypothesizing that these younger stallions (ages ranging from 3 to 4 years) were more at risk of declining fertility with book size than the ‘experienced’ older stallion group (ages ranging from 4 to 13 years). However, the effect of stallion age itself was not assessed in the study. In our study, the proportion of abortion cases was numerically higher in those stallions covering, on average, more than three times a day; however, the case numbers were low resulting in low study power for this variable suggesting value in assessing the variable again with greater case numbers.

Due to the large number of stallions in the study, with some breeding fewer than five pregnancies, it was not statistically appropriate to include stallion as a fixed effect in the regression models. The covering stallion was accounted for through inclusion as a random effect, but no significant stallion level variance was found once the stallion’s age was accounted for. Whilst anecdotal reports suggest certain stallions have a higher proportion of abortions in their book than others, this study found no evidence to support this at the population level. This study found that the shuttle status of the stallion had no effect on the risk of abortion. Shuttling a stallion has been found to have no effect on the seasonal live foal rates for individuals (Pickett & Voss 1998) nor their year-to-year fertility even following up to 10 consecutive shuttling seasons (Walbornn *et al.* 2017).

Limitations in the data collection of some of the exposure variables included in this analysis may affect the validity of the findings. Data from the national stud book were used to collate information on the age at which the mare entered stud in the UK, her reproductive history and stallion book size. The Weatherbys mare return data utilized to determine abortion history is self-reported, likely resulting in an underestimation of mares who suffered an abortion compared to pregnancies which resulted in live foals, due to a negative confirmation bias. Similarly, the exposure variables measuring the age at which the mare entered stud and their reproductive history will only account for UK seasons, therefore, underestimating years for mares who have begun their breeding career abroad. When comparing to previous risk factor cohort studies, there are discrepancies in status proportions, most notably in the barren and rested groups. The proportion of barren or rested mares in the current study cohort was considerably lower than in previous international risk factor studies (Hemberg *et al.* 2004, Nath *et al.* 2010, Hanlon *et al.* 2012, Lane *et al.* 2016) although like Allen *et al.* (2007) in the UK, 17.5% compared with 20%. It is therefore worth considering the populations when comparing data between other regions and cohorts in any future analyses.

In summary, this first multivariable analysis of risk factors for abortion in mares revealed three factors uniquely associated with a modified risk of abortion in this UK Thoroughbred population and an additional factor shared with failure to conceive and EPL. The increased risk after two prior abortions suggests an intrinsic maternal factor, such as an anatomical or genetic predisposition, is key to the development of the condition. To a lesser extent, both conceiving multiple pregnancies and failing to conceive the pregnancy on the first estrous cycle the mare was covered on were associated with an increased risk of abortion. Numerous prior studies have found no association between multiple conceptions and EPL; therefore, this finding is notable. The underlying reason warrants exploration in future studies, including a review of embryo reduction practices. Failure to conceive the pregnancy on the first estrous cycle will reflect a level of subfertility in the mare, either due to failure to conceive or an EEL/EPL and suggests shared pathologies with conception and early pregnancy, such as uterine health. This study found no association with mare age, status, parity or number of previous live foals with abortion. Increasing stallion age was associated with a decreasing risk of abortion in this study; whilst this is contradictory to human and breeding efficiency studies, it likely reflects a healthy worker effect as opposed to a physiological reasoning. Mares who have suffered two or more losses warrant increased monitoring by veterinary surgeons. These mares may benefit from breeding to older stallions. Whilst the results of this study have raised questions for future avenues of research, it also provides tangible data to be implemented to modify clinical and management decisions to improve breeding efficiency and welfare.

## Supplementary Material

Supplement 1: Descriptive results of A. Mare, B. Pregnancy, C. Extrinsic and D. Stallion exposure variables and the proportion of the pregnancies where an abortion (pregnancy loss between day 70 and 300 of gestation) occurred in a cohort of UK Thoroughbreds (n=4,439 pregnancies) *greater than 25% missing data

Supplement 2: Summary of the proportion of missing data in exposure variables collected for analysis of risk factors associated with pregnancy loss after day 70 of gestation in a cohort of UK Thoroughbreds (n=4,439)

Supplement 3: Univariable analysis results of A. Mare, B. Pregnancy, C. Extrinsic and D. Stallion exposure variables associated with abortion in a cohort of UK Thoroughbreds (n=4,439 pregnancies). Mare nested in mare farm were included as random effects (p=0.09) *Likelihood ratio test, **Greater than 25% missing data

## Declaration of interest

A M de Mestre is an Associate Editor for *Reproduction and Fertility* but was not involved in the peer review process of this article. The other authors have nothing to disclose.

## Funding

This work was kindly supported by the Alborada Trust http://dx.doi.org/10.13039/100008288.

## Author contribution statement

J M Roach: Conceptualization, formal analysis, investigation, methodology, project administration, validation, visualization; Roles/Writing – original draft, Writing – review and editing. J C Arango-Sabogal: Formal analysis, methodology, validation, visualization; Writing – review and editing. K C Smith: Funding acquisition, supervision. A K Foote: Funding acquisition, supervision, resources. K L Verheyen: Conceptualization, funding acquisition, methodology, software, supervision; Writing – review and editing. A M de Mestre: Conceptualization, funding acquisition, methodology, resources, supervision; Writing – review and editing.
