# Supplementary material for: Multivariable analysis to determine risk factors associated with abortion in mares
Source: Reprod Fertil. 2022 Nov 14;3(4):301–12. doi: 10.1530/RAF-22-0087 (PMC9782406; doi:10.1530/RAF-22-0087)
Supplement: Supplement 1: Descriptive results of A. Mare, B. Pregnancy, C. Extrinsic and D. Stallion exposure variables and the proportion of the pregnancies where an abortion (pregnancy loss between day 70 and 300 of gestation) occurred in a cohort of UK Thoroughbreds (n=4,439 pregnancies) *greater than 25% mi [file supplementary_table_1.pdf]

Supplement 1: Descriptive results of A. Mare, B. Pregnancy, C. Extrinsic and D. Stallion exposure variables and the proportion of the pregnancies where an abortion (pregnancy loss between day 70 and 300 of gestation) occurred in a cohort of UK Thoroughbreds (n=4,439 pregnancies) *greater than 25% missing data

A.

Variable	Category	Abortion cases (n)	N total	% aborted	95% Confidence Interval
**Mare age (years)**	3	2	60	3.33	0.41, 11.53
	4	12	510	2.35	1.22, 4.07
	5	17	542	3.14	1.84, 4.97
	6	14	542	2.58	1.42, 4.30
	7	13	483	2.69	1.44, 4.56
	8	19	423	4.49	2.73, 6.93
	9	21	372	5.65	3.53, 8.50
	10	10	300	3.33	1.61, 6.04
	11	10	247	4.05	1.96, 7.32
	12	8	214	3.74	1.63, 7.23
	13	6	173	3.47	1.28, 7.40
	14	6	134	4.48	1.66, 9.49
	15	6	107	5.61	2.09, 11.81
	16	3	99	3.03	0.63, 8.60
	17	3	79	3.80	0.79, 10.70
	18	5	61	8.20	2.72, 18.10
	19	5	55	9.09	3.02, 19.95
	20	1	21	4.76	0.12, 23.82
	21	1	9	11.11	0.28, 48.25
	22	1	6	16.67	0.42, 64.12
	24	0	1	0.00	0.00, 97.50
**Status**					
	Maiden	19	761	2.50	1.51, 3.87
	Foaling	108	2,904	3.72	3.06, 4.47
	Barren	31	592	5.24	3.59, 7.35
	Rested	5	182	2.75	0.90, 6.29
**Age of mare at first breeding season (years)**					
	<4	10	161	6.21	3.02, 11.13
	4-7	128	3,240	3.95	3.31, 4.68
	≥8	2	29	6.90	0.85, 22.77
**Number of previous abortions (n)**					
	Maiden	19	761	2.50	1.51, 3.87
	Bred never aborted	100	2,617	3.82	3.12, 4.63
	One previous abortion	15	259	5.79	3.28, 9.37
	Two or more previous abortions	10	50	20.00	10.03, 33.72

Variable	Category	Abortion cases (n)	N total	% aborted	95% Confidence Interval
**Number of previous live foals (n)**					
	0	21	807	2.60	1.62, 3.95
	1	22	672	3.27	2.06, 4.91
	2	19	566	3.36	2.03, 5.19
	3	25	496	5.04	3.29, 7.35
	4	12	436	2.75	1.43, 4.76
	5	18	357	5.04	3.02, 7.85
	6	11	260	4.23	2.13, 7.44
	7	6	211	2.84	1.05, 6.09
	8	6	171	3.51	1.30, 7.48
	9	5	115	4.35	1.43, 9.85
	10	5	84	5.95	1.96, 13.35
	11	5	72	6.94	2.29, 15.47
	12	1	39	2.56	0.06, 13.48
	13	0	24	0.00	0.00, 14.25
	14	3	9	33.33	7.49, 70.07
	15	0	6	0.00	0.00, 45.93
	16	0	3	0.00	0.00, 70.76
	17	0	2	0.00	0.00, 84.19
**Total number of years covered (years)**					
	0	17	571	2.98	1.74, 4.72
	1	14	473	2.96	1.63, 4.92
	2	15	406	3.69	2.08, 6.02
	3	19	325	5.85	3.56, 8.98
	4	9	318	2.83	1.30, 5.30
	5	13	279	4.66	2.50, 7.84
	6	12	232	5.17	2.70, 8.86
	7	6	172	3.49	1.29, 7.44
	8	5	151	3.31	1.08, 7.56
	9	7	127	5.51	2.24, 11.03
	10	4	92	4.35	1.20, 10.76
	11	6	72	8.33	3.12, 17.26
	12	3	70	4.29	0.89, 12.02
	13	2	52	3.85	0.47, 13.21
	14	3	39	7.69	1.62, 20.87
	15	3	30	10.00	2.11, 26.53
	16	0	14	0.00	0.00, 23.16
	17	0	5	0.00	0.00, 52.18
	18	1	4	25.00	0.63, 80.59

Variable	Category	Abortion cases (n)	N total	% aborted	95% Confidence Interval
**Mare farm (farm ID)**					
	1	16	372	4.30	2.48, 6.89
	2	50	951	5.26	3.93, 6.87
	3	0	24	0.00	0.00, 14.25
	4	7	240	2.92	1.18, 5.92
	5	1	20	5.00	0.13, 24.87
	6	0	8	0.00	0.00, 36.94
	7	1	60	1.67	0.04, 8.94
	8	0	52	0.00	0.00, 6.85
	9	15	371	4.04	2.28, 6.58
	10	0	60	0.00	0.00, 5.96
	11	2	55	3.64	0.44, 12.53
	12	16	295	5.42	3.13, 8.66
	13	3	114	2.63	0.55, 7.50
	14	0	42	0.00	0.00, 8.41
	15	1	29	3.45	0.09, 17.76
	16	2	57	3.51	0.43, 12.11
	17	4	113	3.54	0.97, 8.82
	18	1	21	4.76	0.12, 23.82
	19	0	14	0.00	0.00, 23.16
	20	5	167	2.99	0.98, 6.85
	21	3	93	3.23	0.67, 9.14
	22	1	17	5.88	0.15, 28.69
	23	1	69	1.45	0.04, 7.81
	24	2	115	1.74	0.21, 6.14
	25	0	12	0.00	0.00, 26.46
	26	0	2	0.00	0.00, 84.19
	27	11	486	2.26	1.14, 4.01
	28	21	580	3.62	2.25, 5.48
**Oestrous cycle pregnancy conceived on**					
	First oestrous cycle	91	2,828	3.22	2.60, 3.94
	Subsequent oestrous cycle	48	912	5.26	3.91, 6.92
**Month of cover (month)**					
	February	20	578	3.46	2.13, 5.29
	March	55	1,347	4.08	3.09, 5.28
	April	46	1,297	3.55	2.61, 4.70
	May	34	1,044	3.26	2.27, 4.52
	June or later	8	173	4.62	2.02, 8.91

B.

Variable	Category	Abortion cases (n)	N total	% aborted	95% Confidence Interval
**Multiple conceptus**					
	No	110	3,334	3.30	2.72, 3.96
	Yes	29	577	5.03	3.39, 7.14
**Fetal sex**					
	Female	28	1,946	1.44	0.96, 2.07
	Male	30	2,098	1.43	0.97, 2.04
**Altrenogest administered during gestation***					
	No	45	1,688	2.67	1.95, 3.55
	Yes	3	122	2.46	0.51, 7.02

C.

Variable	Category	Abortion cases (n)	N total	% aborted	95% Confidence Interval
**Mare travelled following cover**					
	No	123	3,499	3.52	2.93, 4.18
	Yes	37	839	4.41	3.12, 6.03
**Year of cover (year)**					
	2013	31	1,145	2.71	1.85, 3.82
	2014	29	957	3.03	2.04, 4.32
	2015	35	762	4.59	3.22, 6.33
	2016	28	787	3.56	2.38, 5.10
	2017	40	788	5.08	3.65, 6.85

D.

Variable	Category	Abortion cases (n)	N total	% aborted	95% Confidence Interval
**Stallion age (years)**					
	3	1	5	20.00	0.51, 71.64
	4	11	152	7.24	3.67, 12.58
	5	20	477	4.19	2.58, 6.40
	6	25	427	5.85	3.82, 8.52
	7	10	202	4.95	2.40, 8.92
	8	9	329	2.74	1.26, 5.13
	9	10	320	3.13	1.51, 5.67
	10	10	203	4.93	2.39, 8.87
	11	7	309	2.27	0.92, 4.61
	12	11	299	3.68	1.85, 6.49
	13	9	303	2.97	1.37, 5.56
	14	10	324	3.09	1.49, 5.60
	15	10	291	3.44	1.66, 6.23
	16	2	153	1.31	0.16, 4.64
	17	6	131	4.58	1.70, 9.70
	18	3	92	3.26	0.68, 9.23
	19	3	87	3.45	0.72, 9.75
	20	2	150	1.33	0.16, 4.73
	21	2	67	2.99	0.36, 10.37
	22	0	37	0.00	0.00, 9.49
	23	1	27	3.70	0.09, 18.97
	24	0	23	0.00	0.00, 14.82
	25	0	1	0.00	0.00, 97.50
**Book size (average number of breedings per day) (n)**					
	Up to every other day	4	153	2.61	0.72, 6.56
	Up to once a day	11	380	2.89	1.45, 5.12
	Up to twice a day	99	2,697	3.67	2.99, 4.45
	Up to three covers a day	43	1,123	3.83	2.78, 5.12
	Greater than three covers a day	4	55	7.27	2.02, 17.59
**Stallion shuttled season prior**					
	No	128	3,432	3.73	3.12, 4.42
	Yes	32	914	3.50	2.41, 4.91

Variable	Category	Abortion cases (n)	N total	% aborted	95% Confidence Interval
**Stallion farm (farm ID)**					
	F01	8	164	4.88	2.13, 9.39
	F02	51	1,307	3.90	2.92, 5.10
	F03	0	55	0.00	0.00, 6.49
	F04	6	220	2.73	1.01, 5.84
	F09	19	651	2.92	1.77, 4.52
	F11	1	19	5.26	0.13, 26.03
	F12	9	263	3.42	1.58, 6.40
	F20	0	52	0.00	0.00, 6.85
	F29	28	670	4.18	2.79, 5.98
	F36	3	40	7.50	1.57, 20.39
	F37	0	1	0.00	0.00, 79.50
	F42	0	2	0.00	0.00, 84.19
	F50	15	463	3.24	1.82, 5.29
	F51	0	1	0.00	0.00, 97.50
	F52	5	65	7.69	2.54, 17.05
	F53	1	57	1.75	0.04, 9.39
	F54	0	3	0.00	0.00, 70.76
	F55	3	28	10.71	2.27, 28.23
	F56	3	91	3.30	0.69, 9.33
	F57	1	8	12.50	0.32, 52.65
	F58	0	4	0.00	0.00, 60.24
	F59	0	2	0.00	0.00, 84.19
	F60	0	11	0.00	0.00, 28.49
	F61	0	2	0.00	0.00, 84.19
	F62	1	12	8.33	0.21, 38.48
	F64	0	28	0.00	0.00, 12.34
	F65	0	5	0.00	0.00, 52.18
	F66	2	36	5.56	0.68, 18.66
	F67	2	23	8.70	1.07, 28.04
	F68	0	1	0.00	0.00, 97.50
	F69	3	60	5.00	1.04, 13.92
	F70	0	15	0.00	0.00, 21.80
