# Supplementary material for: Multivariable analysis to determine risk factors associated with abortion in mares
Source: Reprod Fertil. 2022 Nov 14;3(4):301–12. doi: 10.1530/RAF-22-0087 (PMC9782406; doi:10.1530/RAF-22-0087)
Supplement: Supplement 2: Summary of the proportion of missing data in exposure variables collected for analysis of risk factors associated with pregnancy loss after day 70 of gestation in a cohort of UK Thoroughbreds (n=4,439) [file supplementary_table_2.pdf]

Supplement 2: Summary of the proportion of missing data in exposure variables collected for analysis of risk factors associated with pregnancy loss after day 70 of gestation in a cohort of UK Thoroughbreds (n=4,439)

	Exposure variable	Number of pregnancies with missing data (n)	Proportion of pregnancies with missing data (%)
Mare factors	Mare	0	0.0
	Mare age	1	0.0
	Mare farm	0	0.0
	Status	0	0.0
	Age of mare at first breeding season	1,035	23.3
	Number of previous live foals	109	2.5
	Number of previous abortions	773	17.4
	Total number of years covered	1,032	23.2
	Month of cover	0	0.0
	Estrous cycle pregnancy conceived on	702	15.8
Pregnancy factors	Multiple conceptus	539	12.1
	Fetal Sex	452	10.2
	Altrenogest administered during gestation	2,649	59.7
Extrinsic factors	Mare travelled following cover	104	2.3
	Year of cover	0	0.0
Stallion factors	Stallion	11	0.2
	Stallion age	30	0.7
	Stallion farm	82	1.8
	Book size	31	0.7
	Shuttled season prior	95	2.1
Foaling mare factors*	Foaling Date	31	1.1
	Foaling Month	31	1.1
	Days from foaling to cover	82	2.8
	Foal heat cover	32	1.1

*Calculated from the sub-population of mares with a foaling status (n=2,945)
