# Supplementary material for: Multivariable analysis to determine risk factors associated with abortion in mares
Source: Reprod Fertil. 2022 Nov 14;3(4):301–12. doi: 10.1530/RAF-22-0087 (PMC9782406; doi:10.1530/RAF-22-0087)
Supplement: Supplement 3: Univariable analysis results of A. Mare, B. Pregnancy, C. Extrinsic and D. Stallion exposure variables associated with abortion in a cohort of UK Thoroughbreds (n=4,439 pregnancies). Mare nested in mare farm were included as random effects (p=0.09) *Likelihood ratio test, **Greater tha [file supplementary_table_3.pdf]

Supplement 3: Univariable analysis results of A. Mare, B. Pregnancy, C. Extrinsic and D. Stallion exposure variables associated with abortion in a cohort of UK Thoroughbreds (n=4,439 pregnancies). Mare nested in mare farm were included as random effects (p=0.09) *Likelihood ratio test, **Greater than 25% missing data

A.

Variable	Category	Odds ratio	95% Confidence Interval	Wald P value	LRT* P value
**Mare age (years)**		1.06	1.02, 1.11	0.002	0.002
**Status**					0.11
	Maiden	*Reference*			
	Foaling	1.53	0.93, 2.53	0.10	
	Barren	2.06	1.13, 3.77	0.02	
	Rested	1.11	0.40, 3.05	0.85	
**Age of mare at first breeding season (years)**					0.32
	<4	1.66	0.81, 3.40	0.16	
	4-7	*Reference*			
	≥8	1.61	0.34, 7.65	0.55	
**Number of previous live foals (n)**		1.06	1.00, 1.11	0.03	0.03
**Number of previous abortions (n)**					<0.001
	Maiden	*Reference*			
	Bred never aborted	1.55	0.94, 2.55	0.09	
	One previous abortion	2.39	1.18, 4.84	0.02	
	Two or more previous abortions	9.92	4.25, 23.13	<0.001	
**Total number of years covered (years)**		1.05	1.01, 1.10	0.02	0.02
**Oestrous cycle pregnancy conceived on**					0.01
	First oestrous cycle covered on	*Reference*			
	Subsequent oestrous cycle covered on	1.63	1.12, 2.37	0.01	
**Month of cover (month)**					0.83
	February	*Reference*			
	March	1.20	0.70, 2.06	0.51	
	April	1.05	0.60, 1.83	0.86	
	May	0.96	0.54, 1.73	0.90	
	June or later	1.38	0.57, 3.31	0.47	

B.

Variable	Category	Odds ratio	95% Confidence Interval	Wald P value	LRT* P value
**Multiple conceptus**					0.03
	No	*Reference*			
	Yes	1.64	1.06, 2.54	0.03	
**Fetal sex**					0.96
	Female	*Reference*			
	Male	0.99	0.59, 1.66	0.96	
**Altrenogest administered during gestation****					0.89
	No	*Reference*			
	Yes	0.92	0.28, 3.01	0.89	

C.

Variable	Category	Odds ratio	95% Confidence Interval	Wald P value	LRT* P value
**Mare travelled following cover**	No	*Reference*			0.34
	Yes	1.22	0.81, 1.83	0.34	
**Year of cover (year)**	2013	*Reference*			0.05
	2014	1.14	0.67, 1.93	0.63	
	2015	1.74	1.04, 2.91	0.03	
	2016	1.34	0.78, 2.29	0.29	
	2017	1.98	1.19, 3.28	0.01	

D.

Variable	Category	Odds ratio	95% Confidence Interval	Wald P value	LRT* P value
**Stallion age (years)**		0.94	0.91, 0.98	0.001	0.001
**Book size (Average number of breedings per day)**					0.63
	Up to every other day	0.73	0.26, 2.09	0.56	
	Up to once a day	0.80	0.42, 1.55	0.51	
	Up to twice a day	*Reference*			
	Up to three covers a day	1.06	0.72, 1.55	0.76	
	Greater than three covers a day	2.04	0.68, 6.10	0.20	
**Stallion shuttled season prior**					0.66
	No	*Reference*			
	Yes	0.91	0.60, 1.38	0.66	

Variable	Category	Odds ratio	95% Confidence Interval	Wald P value	LRT* P value
**Stallion farm (farm ID)**					0.54
	F01	1.41	0.62, 3.22	0.41	
	F02	*Reference*			
	F03				
	F04	0.81	0.30, 2.15	0.67	
	F09	0.78	0.43, 1.43	0.43	
	F11	1.52	0.17, 13.49	0.71	
	F12	0.96	0.44, 2.10	0.91	
	F20				
	F29	1.20	0.72, 2.01	0.49	
	F36	2.27	0.62, 8.40	0.22	
	F37				
	F42				
	F50	0.76	0.41, 1.41	0.38	
	F51				
	F52	2.51	0.87, 7.21	0.09	
	F53	0.49	0.06, 3.72	0.49	
	F54				
	F55	3.53	0.90, 13.92	0.07	
	F56	0.81	0.24, 2.78	0.74	
	F57	5.02	0.49, 51.25	0.17	
	F58				
	F59				
	F60				
	F61				
	F62	2.67	0.29, 24.78	0.39	
	F64				
	F65				
	F66	1.66	0.35, 7.76	0.52	
	F67	3.02	0.61, 14.89	0.18	
	F68				
	F69	1.33	0.38, 4.64	0.66	
	F70				
